# Cysteamine-coated gold nanoparticles for bimodal colorimetric detection with inverse sensitivity: a proof-of-concept with lysozyme†

**DOI:** 10.1039/c9ra07930k

**Published:** 2020-01-06

**Authors:** Jing Luen Wai, Siu Yee New

**Affiliations:** School of Pharmacy, University of Nottingham Malaysia Jalan Broga, Semenyih 43500 Selangor Malaysia SiuYee.New@nottingham.edu.my

## Abstract

Cysteamine-coated gold nanoparticles (cysAuNPs) are positively charged as-synthesised and hence can interact with negatively charged DNA with ease. We have investigated the dependency of the particles' dispersion stage on different concentrations of lysozyme-binding aptamer (LBA). On top of the commonly reported phenomenon where cysAuNPs aggregate as the concentration of LBA increases, we observed that cysAuNPs redispersed after the amount of LBA achieved a certain threshold, dubbed as the critical redispersion concentration (CRC). By harnessing the aggregation and dispersion behaviour of cysAuNPs at LBA below and above the CRC, respectively, we have demonstrated a bimodal colorimetric aptasensor to detect lysozyme as a proof-of-concept study. Apart from being able to quantify the lysozyme in different ranges of concentrations with a visual change in colour, this aptasensor also demonstrated a novel concept of inverse sensitivity (*i.e.* higher signal with less analyte), leading to a 24-fold higher of signal-to-noise ratio (SNR), in comparison to the conventional sensors. The aptasensor can also selectively distinguish lysozyme and eliminate false results from other control proteins *via* both modes. The generalisability, as well as potential of cysAuNPs for bimodal colorimetric detection and inverse sensitivity behaviour have made this material an interesting alternative to citrate-coated AuNPs.

## Abstract 2

An aptasensor derived from cysteamine-gold nanoparticles for bimodal colorimetric detection and with inverse sensitivity pattern.

## Graphical abstract

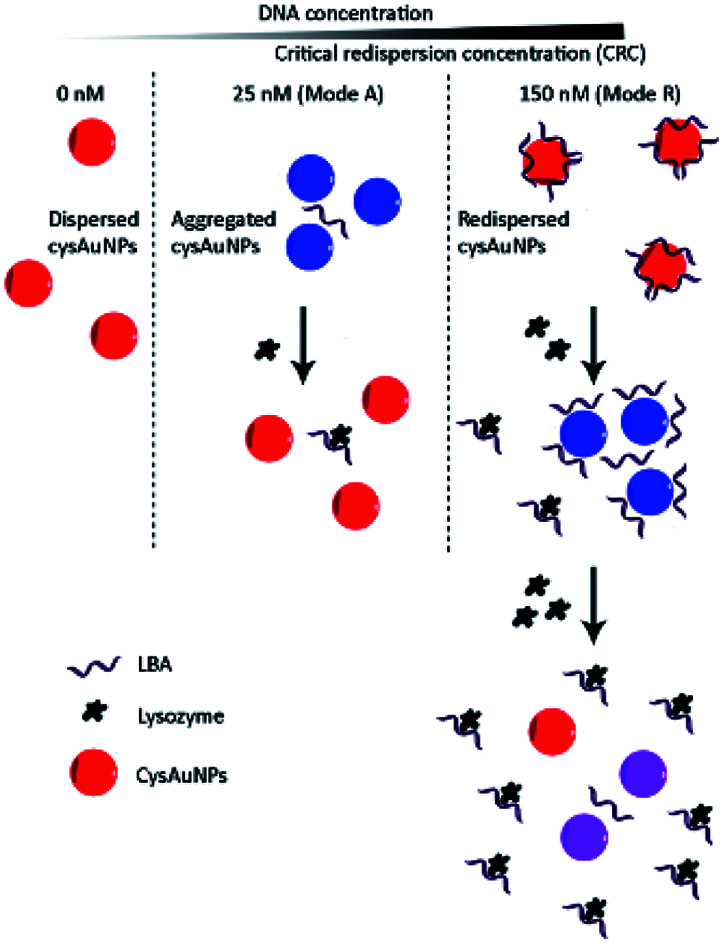

## Introduction

Gold nanoparticles (AuNPs) have received substantial attention due to their unique and tuneable optoelectronic properties that mainly arises from surface plasmon resonance (SPR). Briefly, AuNPs exhibiting SPR change their appearance colour from ruby red to bluish grey, depending on their overall sizes as well as dispersion-aggregation states. In order to avoid undesirable agglomeration, AuNPs are typically stabilised by ligands during synthesis. Among these, citrate-stabilised AuNPs (citAuNPs) are the most popular type, with the citrate ligands bound to the gold surface *via* monocarboxylate bridging, dicarboxylate bridging and monocarboxylate monodentate modes^1^ that provide electrostatic repulsion. Since the binding energy of Au-carboxylate is weak, the citrate layer could be easily disrupted by either ionic or pH changes, resulting in unstable colloids. To make the nanomaterials more suitable for biosensing, most studies replace the citrate layer with thiolated ligands (especially thiolated DNA) that form stronger covalent Au–S bonds and confer specific functionality. Typically, such modification involves salt aging technique developed by Mirkin and co-workers.^2^ However, the protocol *per se* is laborious and time-consuming due to undesired electrostatic repulsion between both negatively charged citAuNPs and DNA that has to be screened off delicately.^3^ With that in regards, considerable efforts have been devoted in modulating the interaction between DNA and AuNPs to achieve surface functionalisation. For instance, Hsing and co-workers presented a new method to synthesise DNA-functionalised AuNPs *via* mononucleotide-mediated conjugation.^4^ In later years, Liu and co-workers introduced pH-assisted^5,6^ and freezing-directed^7^ routes to prepare DNA-modified citAuNPs. However, excessive reagents or drastic changes in reaction conditions (*i.e.* pH or temperature) are required, which can be unfavourable for downstream applications.

This study aims to address the aforementioned limitations by studying non-citrate AuNPs, *i.e.* cysteamine-stabilised AuNPs (cysAuNPs) in details. Being the shortest aminothiol, cysteamine offers both thiol and amine groups that enable strong binding to gold with a net positive surface charge.^8,9^ Hence, cysAuNPs can interact directly with anionic DNA without the need of salt addition. While the simplicity of preparation and generality of cysAuNPs are comparable to that of citAuNPs, the former is relatively stable due to the stronger Au–S bonds as previously described.^10,11^ Given their interesting properties, cysAuNPs have been incorporated into various assays for different sensing purposes, including colorimetric^8,12–20^ and electrochemical.^21,22^ In the context of cysAuNPs-derived aptasensors, they are mainly based on the following general principles:

(i) In the absence of targets, electrostatic interaction between oppositely charged DNA aptamers and cysAuNPs leads to close proximity of the particles and aggregate them;

(ii) In the presence of targets, the specific recognition between DNA aptamers and targets would negate the electrostatic interaction between the aptamers and cysAuNPs, leading to dispersed particles.

**Scheme 1:**
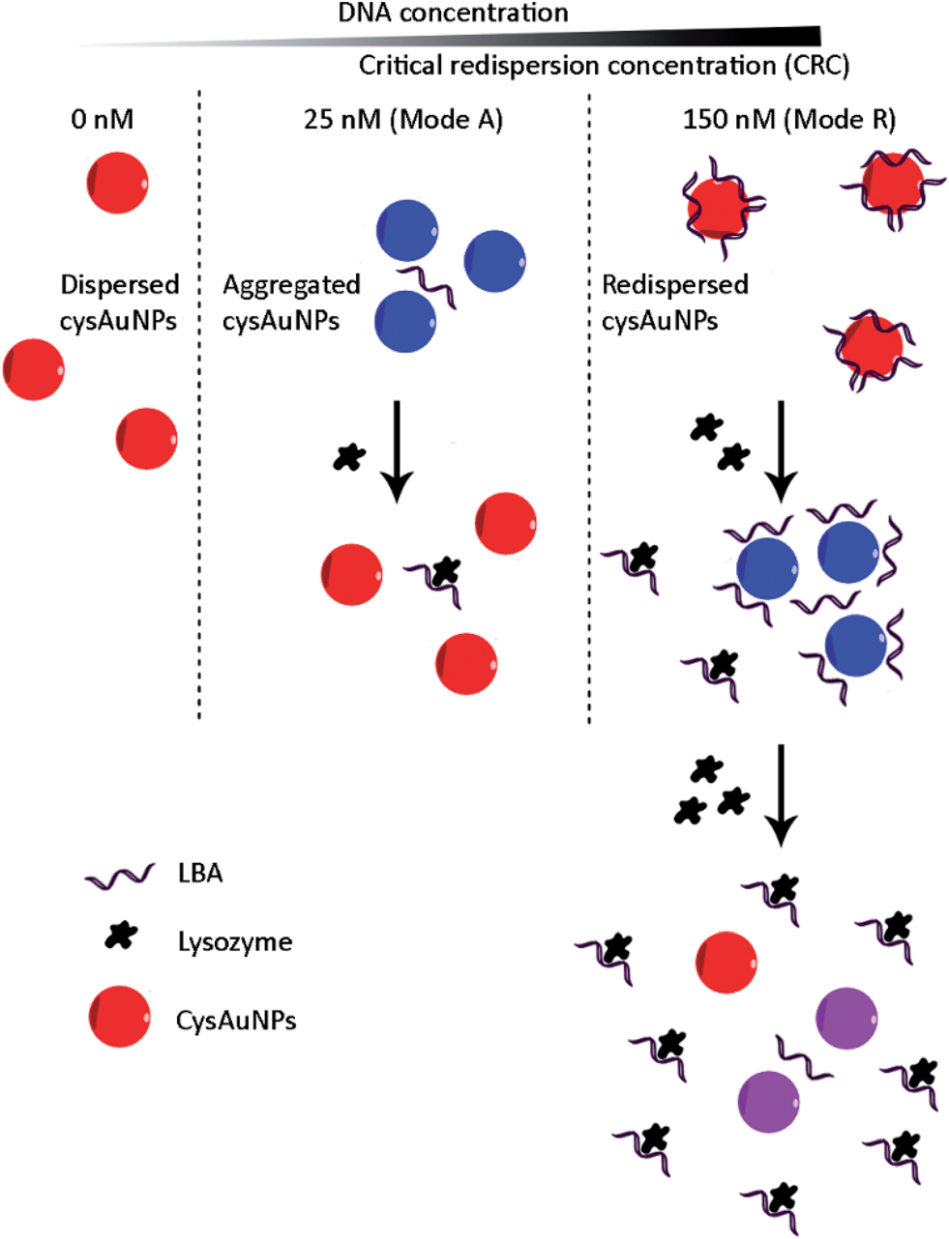
Proposed cysAuNPs-based aptasensor with bimodal responses (modes A and R).

Keeping this in mind, we took the concept one step further by systematically measuring the dispersion of cysAuNPs over a wide range of DNA aptamer concentrations, with lysozyme-binding aptamer (LBA) as a model. On top of the usual observation where cysAuNPs aggregated in the presence of LBA, we found that the particles redispersed after LBA reached a critical redispersion concentration (CRC), attributed to the formation of anionic LBA layer surrounding cysAuNPs. This inspires us to develop a bimodal assay to detect lysozymes by modulating the concentration of LBA (Scheme 1):

(i) Mode A (A = aggregation), where concentration of LBA that could aggregate cysAuNPs was employed. The presence of targets (*i.e.* lysozymes) would reduce the aggregation of cysAuNPs as aforementioned;

(ii) Mode R (R = redispersion) with excess LBA at CRC regime. Adding lysozymes to the redispersed cysAuNPs would lead to two observations: (1) formation of lysozyme/LBA complexes would deplete the free LBA adsorbed onto the particle surface, subsequently cysAuNPs aggregate due to electrostatic masking; (2) further increased amount of lysozyme would result in exhausted LBA layers, hence cysAuNPs re-establish their positive charges and repel each other.

To the best of our knowledge, DNA concentration-dependent bimodal response has never been reported on non-modified spherical AuNPs for colorimetric detection. Herein, we show that not only the bimodal nature has increased the assay accuracy, but it also spans a wide dynamic range with each mode covers a different range. Noteworthily, the assay in mode R can even detect lysozyme in an inverse sensitivity pattern with exceptional signal-to-noise ratio (SNR). Given the fast and direct electrostatic interaction with DNA, the proposed aptasensor also avoids the otherwise time-consuming surface-functionalisation of AuNPs and enables a near immediate response. Hence, this study proposes an alternative to the conventional citAuNPs through demonstrating the possibility of cationic cysAuNPs in the sensing field.

## Experimental

### Chemicals and materials

Lysozyme, cytochrome C, insulin, vascular endothelial growth factor (VEGF), bovine serum albumin (BSA) and gold(iii) chloride (HAuCl_4_) solution were purchased from Sigma-Aldrich (St. Louis, USA). 2-Aminoethanethiol or cysteamine (C_2_H_7_NS) was obtained from Tokyo Chemical Industry Co., Ltd (Kyoto, Japan). Sodium borohydride (NaBH_4_) was purchased from Nacalai Tesque Inc. (Kyoto, Japan). The oligonucleotide was provided by Integrated DNA Technologies (IDT). 1× phosphate buffered saline (PBS) at pH 7.4 was used. All reagents and chemicals were of analytical grade and used without further purification. Deionised water (DI H_2_O) with measured conductivity of 18.2 MΩ cm was used throughout all experiments. Unless otherwise stated, all experiments were conducted at room temperature (20 °C).

### Preparation of oligonucleotide

The oligonucleotide of LBA with sequence of 5′- ATC TAC GAA TTC ATC AGG GCT AAA GAG TGC AGA GTT ACT TAG -3′, was briefly centrifuged and reconstituted to a recommended volume in DI H_2_O. The LBA stock solution was then stored at −20 °C until use. Before each experiment, the LBA concentration was estimated through UV-Vis spectrophotometry.

### Preparation of cysAuNPs

All glassware was rinsed with aqua regia (HNO_3_ : HCl of 3 : 1 (v/v)), then washed extensively with DI H_2_O and air-dried overnight. CysAuNPs were prepared according to published protocols with slight modifications.^10^ In brief, 400 μL of cysteamine (213 mM) was mixed with 40 mL of HAuCl_4_ (1.42 mM) solution under gentle stirring in dark. After that, 10 μL of fresh cold NaBH_4_ (10 mM) was added under vigorous stirring for another 10 min, followed by mild stirring for 30 min. The solution was then stored in dark at room temperature for overnight before use. All cysAuNPs were used within two months.

### Instrumental analysis

UV-Vis absorbance spectra were recorded on an Epoch™ microplate spectrophotometer (Biotek Instrument, USA). DNA quantification was performed using a NanoDrop™ One (Nanodrop, USA). Dynamic light scattering and zeta potential analyses were conducted using a Malvern Nanoseries Zetasizer (Malvern, UK). Scanning-transmission electron microscopy (STEM) images were taken on a Quanta 400F (FEI Company, USA) at 15 kV under high vacuum, with the samples being air-dried on formvar-coated copper grids without specific fixation.

The obtained UV-Vis absorbance spectra were analysed ratiometrically for their dispersion ratios (*D*_r_) by comparing the absorbances at 526 and 700 nm as shown in below:1
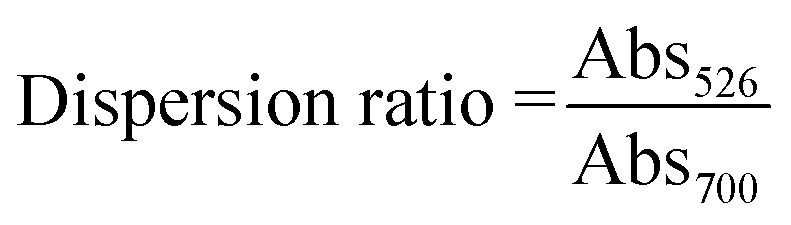


The obtained data were then further analysed for their SNR and limit of detection (LOD) using the equations given below:2
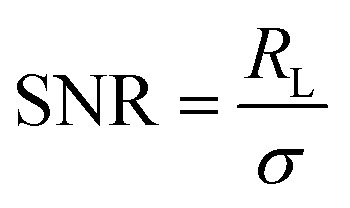
3
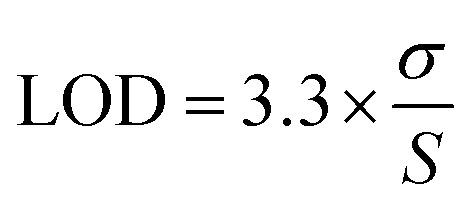
where *R*_L_ = signal response of least known concentration, *σ* = standard deviation of noise of blank sample (no analyte), *S* = slope of the linear regression model obtained.

### Interaction between LBA and cysAuNPs

The LBA was first diluted to the desired concentration in buffer. Then, the LBA was heat treated at 90 °C for 5 min and slowly cooled down to room temperature. In a 96-well plate, 5 μL of heat-treated LBA was incubated with 95 μL of cysAuNPs for 5 min under mild mixing on a rotary shaker. The samples were then photographed and subjected to UV-Vis measurement as previously described.

### Detection of lysozyme using LBA-cysAuNPs aptasensor

Two different concentrations of LBA (25 and 150 nM of final concentrations) were used to detect lysozyme *via* modes A and R, respectively. The LBAs were prepared fresh and heat-treated as mentioned above, then incubated with various concentrations of lysozyme for 1 hour. After that, 5 μL of the mixture was added into 95 μL of cysAuNPs for 5 min in a 96-well plate under mild mixing on a rotary shaker. The samples were then photographed and subjected to UV-Vis absorbance measurement as previously described.

## Results and discussion

### Characterisation of CysAuNPs

**Fig. 1:**
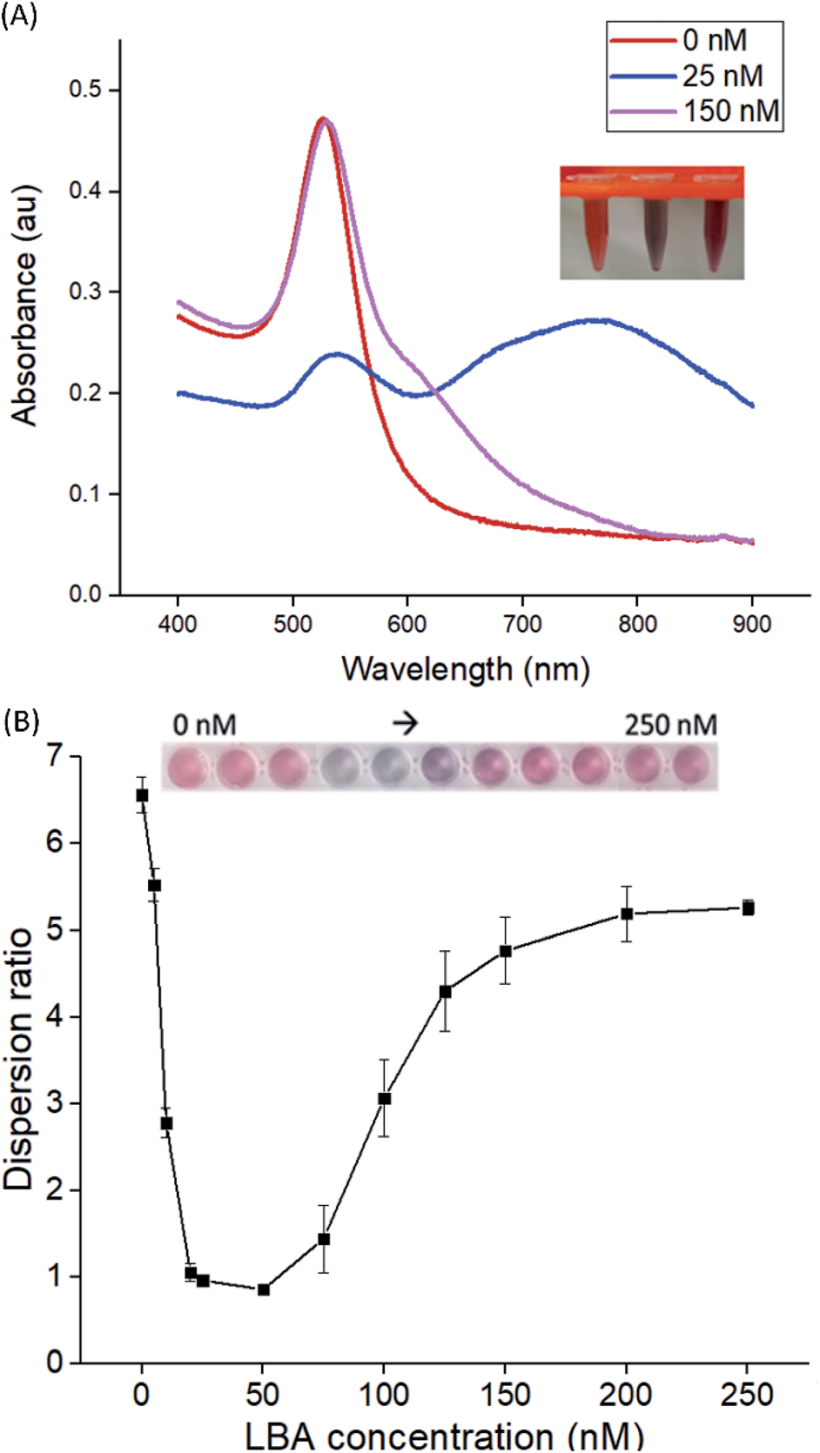
(A) UV-Vis absorbance spectra of cysAuNPs in the absence and presence of different concentrations of LBA. (B) Plot of dispersion ratio of cysAuNPs in the presence of different concentrations of LBA in PBS buffer. Results were presented as mean ± S.D. (*n* = 3). Inset: the corresponding photographs show gradual colour changes of cysAuNPs samples upon mixing with LBA.

CysAuNPs were prepared *via* one-pot synthesis reaction without post-synthesis modification.^23–25^ In accordance with other reports, the as-synthesised cysAuNPs in this study were ruby-red in colour with a characteristic localised SPR (LSPR) band peaked at 526 nm (Fig. 1A).^11,26^ The STEM image reveals homogenously dispersed spherical particles, with an average physical diameter of 28 nm (Table 1). The stability of cysAuNPs is further evidenced from their zeta potential measured at 44.3 mV, implying that the particles are highly dispersed by virtue of electro-repulsive forces among the protonated cysteamine ligands (p*K*_a_ = 8.3). Although cysteamine is expected to cap onto the AuNPs through strong Au–S bond due to the presence of thiol group, there is also possibility of Au–N coordinative bond *via gauche* conformation.^27–29^ To investigate the stability of cysAuNPs in the presence of thiol-containing molecules, we also studied the interference effects of mercaptoacetic acid and acetylcysteine. While mercaptoacetic acid is known to cause severe and irreversible aggregation on citAuNPs,^30^ we have shown that the cysAuNPs could tolerate a high concentration up to 12.5 μM. Similar stability was observed in the presence of acetylcysteine (Fig. S1†).

**Table 1:** Summary of STEM images and zetasizer results of cysAuNPs under different conditions: (A) blank PBS buffer, (B) incubated with 25 nM of LBA and (C) incubated with 150 nM of LBA, respectively. Scale bar = 400 nm

Parameter	Sample		
	(A) Blank cysAuNPs	(B) CysAuNPs with 25 nM LBA	(C) CysAuNPs with 150 nM LBA
STEM image (scale bar = 200 nm)	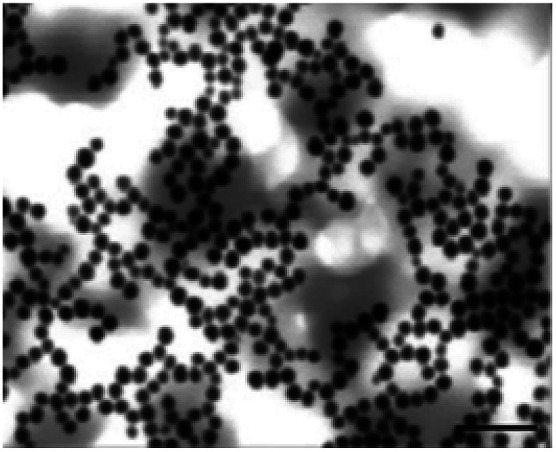	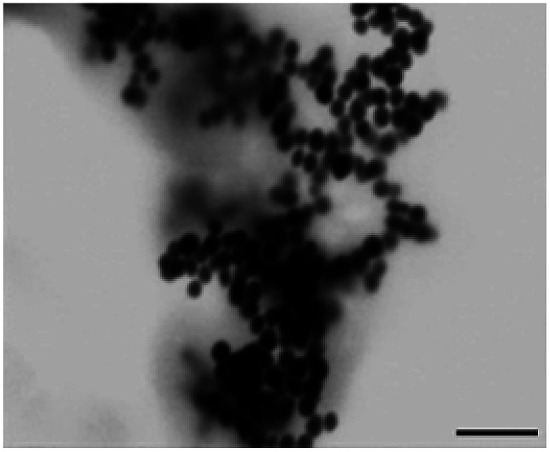	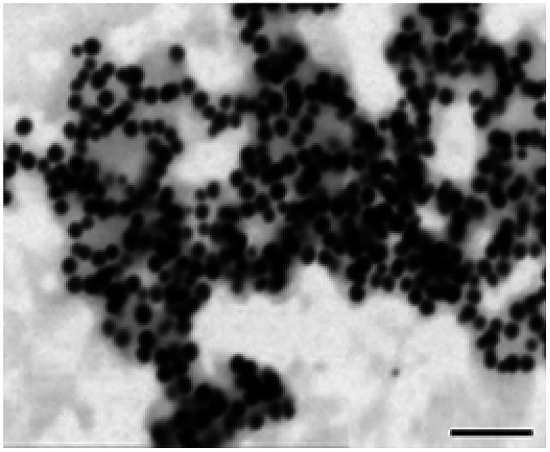
Hydrodynamic size (nm)	45.52 ± 0.41	Severely aggregated	46.33 ± 0.41
PDI	0.15 ± 0.01	Severely aggregated	0.35 ± 0.05
Zeta potential (mV)	44.3 ± 1.37	17.5 ± 1.95	−21.6 ± 2.10

### Interaction between LBA and CysAuNPs

LBA used in this study was reported by Cox *et al.*, with a dissociation constant of 31 nM for lysozyme/LBA complex.^31^ Similar as other ssDNA, the polyanionic LBA wraps on cysAuNPs *via* electrostatic interaction at pH 7.4. As the amount of LBA increases and the interparticle distance reduces, the LSPR band red shifts. Such changes are coupled with a gradual decrease in dispersion and a colour transition of colloidal solution from red to bluish grey (Fig. 1 and S2†). The zeta potential of cysAuNPs in the presence of 25 nM LBA (where cysAuNPs aggregated and appeared blue) shows significant reduction from 44.3 to 17.5 mV, explaining the destabilisation of the colloidal system. As the amount of LBA increases to 50 nM and above, cysAuNPs change from aggregation to dispersion, accompanying with an obvious colour changing from blue to red. We termed this concentration of LBA as “critical redispersion concentration” (CRC), wherein any concentration of LBA at and above CRC would lead to redispersion of cysAuNPs. The zeta potential of cysAuNPs with LBA at CRC regime (*i.e.* 150 nM, where the particles redispersed and appeared red) was −21.6 mV, implying that excess LBA adsorbs onto the surface of cysAuNPs and renders them negatively charged. Additionally, the hydrodynamic particle size and STEM image of cysAuNPs at CRC regime are similar to that of blank particles, further confirming the retained stability of cysAuNPs.

### Aptasensor for bimodal detection of lysozyme

**Fig. 2:**
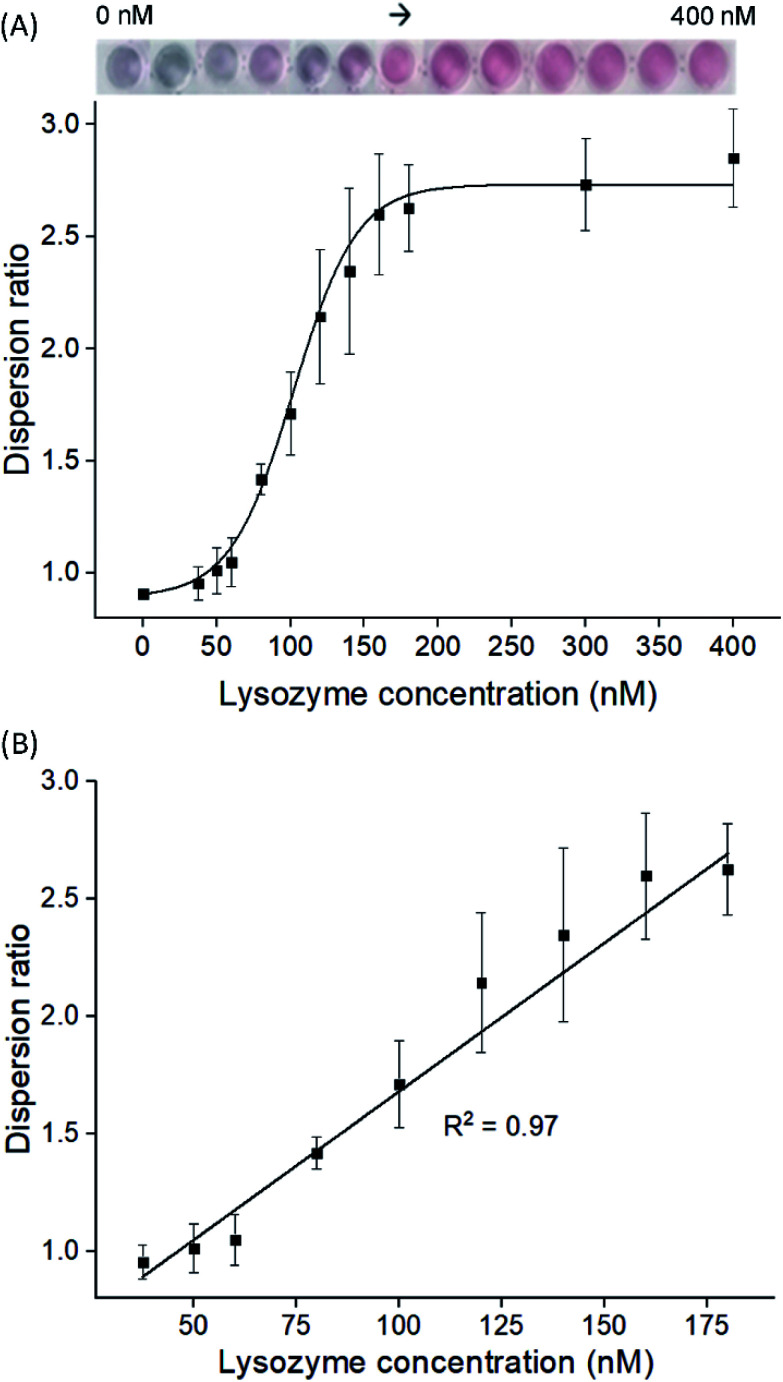
(A) Dispersion ratio plot of cysAuNPs to detect lysozyme at different concentrations *via* mode A with the (B) linear regression fitting model. Results were presented as mean ± S.D (*n* = 3). Inset: the corresponding photographs show gradual colour changes of cysAuNPs samples containing 25 nM of LBA and different amount of lysozyme.

To examine the feasibility of our proposed aptasensor, we have chosen two concentrations of LBA to detect lysozymes *via* modes A and R, respectively. In all modes, the red or blue shift of LSPR band would serve as a quantifiable signal corresponding to the concentration of lysozyme. We first employed LBA at 25 nM, to represent concentration below CRC in mode A, where cysAuNPs aggregated at this stage. In the presence of lysozyme, the LBA binds to its target and forms rigid lysozyme/LBA complexes. This would reduce the amount of negatively charged LBA that interacts electrostatically with cysAuNPs, leading to an increased dispersion (Fig. S3†).^8^Fig. 2 shows that as the concentration of lysozyme increases, a good linear response was observed, accompanied with a colour transition from bluish grey to red (note that due to high amount of samples in a 96-well plate, different photography lighting and angles may influence the overall appearance of some sample images). The linear range was from 37.5 to 180 nM with calculated LOD of 2.29 nM, which is considerably lower than the lysozyme content reported in submandibular saliva (*i.e.* 353–468 nM)^32^ and in colostrum milk (*i.e.* 4.7 μM).^33^

**Fig. 3:**
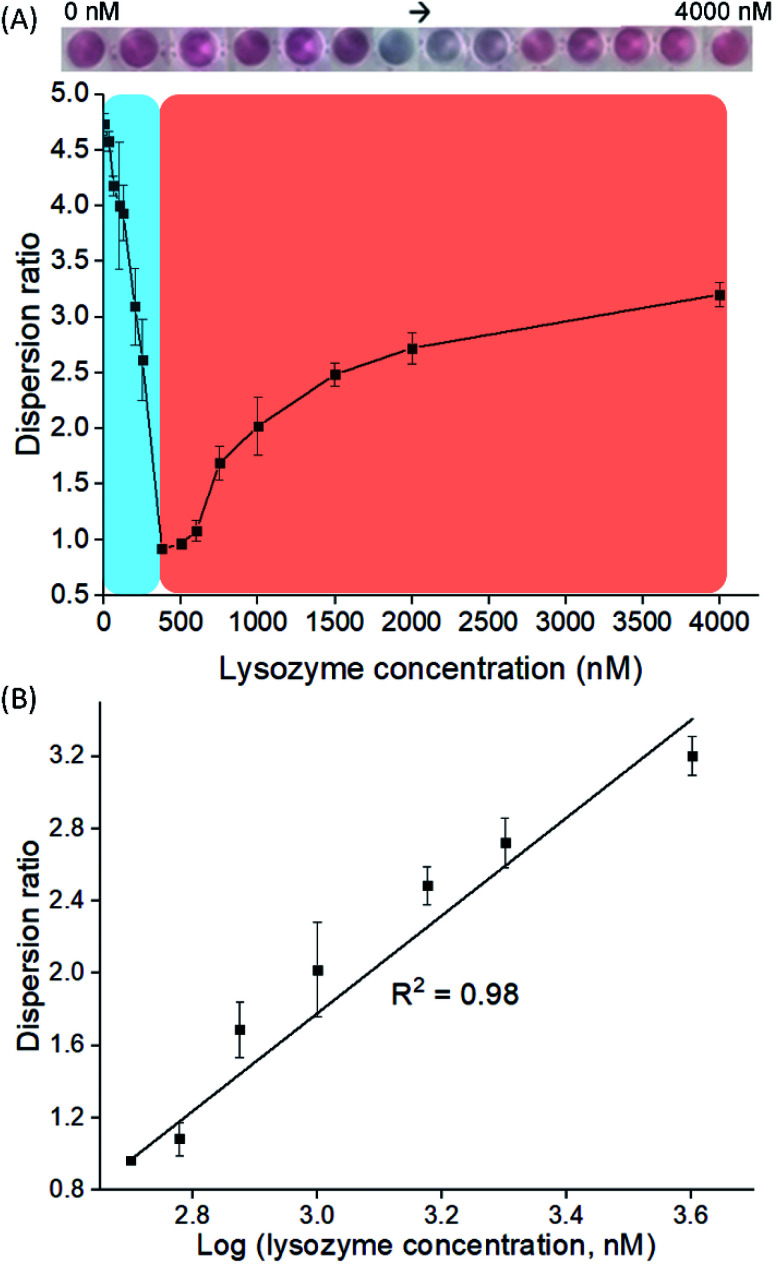
(A) Dispersion ratio plot of cysAuNPs to detect lysozyme at different concentrations *via* mode R with the (B) linear regression fitting model of response in the red regime. Results were presented as mean ± S.D (*n* = 3). Inset: gradual colour changes of cysAuNPs samples containing 150 nM of LBA and different amount of lysozyme.

On the other hand, 150 nM of LBA was used in mode R to represent concentration above CRC, where cysAuNPs were in dispersion stage. In this mode, introducing lysozyme disrupts the electrostatic adsorption of LBA onto cysAuNPs and results in decreased dispersion, as indicated in the blue regime (Fig. 3A and S4†). However, as the concentration of lysozyme further increases, the cysAuNPs response is inverted, showing increasing trend of dispersion ratio (*i.e.* red regime). We reasoned that further formation of lysozyme/LBA complexes has depleted the amount of LBA available to aggregate cysAuNPs. Hence, the particles regain their stability *via* electro-repulsive positive charge conferred by the cysteamine ligands. It is worth noting that such response curve demonstrates an inverse sensitivity behaviour, *i.e.* lower analyte yields higher response. In this case, the dispersion ratio at inverse sensitivity limit (ISL; *i.e.* the observed LOD in red regime at 375 nM) is 0.918, implying the highest aggregation level of nanoparticles. The linearity of response in red regime shows a good *R*^2^ value from 500 to 4000 nM (Fig. 3B). As the blue regime is so narrow and reversed to that in red, response below the ISL is almost negligible. Such phenomenon leads to significantly improved SNR of 72.56, a whopping 24-fold higher than the typically required SNR of 3 at LOD for most sensors.^34^ The novel idea of inverse sensitivity was first proposed by Rodrıguez-Lorenzo *et al.* using enzyme-directed silver deposition on gold nanostars to detect cancer biomarker.^35^ Later on, Pallares *et al.* further developed the concept using hexadecyltrimethylammonium bromide (CTAB)-coated gold nanorods (AuNRs) for quantification of extracellular DNA and circulating cell-free DNA.^36,37^ To the best of our knowledge, this is the first example of inverse sensitivity being demonstrated on spherical AuNPs. While the performance of CTAB-AuNRs could be affected by aspect ratio of the nanorods,^37^ utilising the cysAuNPs *per se* is more controllable considering they are round shaped.

The cysAuNPs-derived aptasensor presents several advantages as compared to other colorimetric aptasensors used for lysozyme detection (Table S1†). This includes versatility of bimodal sensing modes, wide and tuneable detection range, low LOD, as well as exceptional SNR.

### Selectivity test

**Fig. 4:**
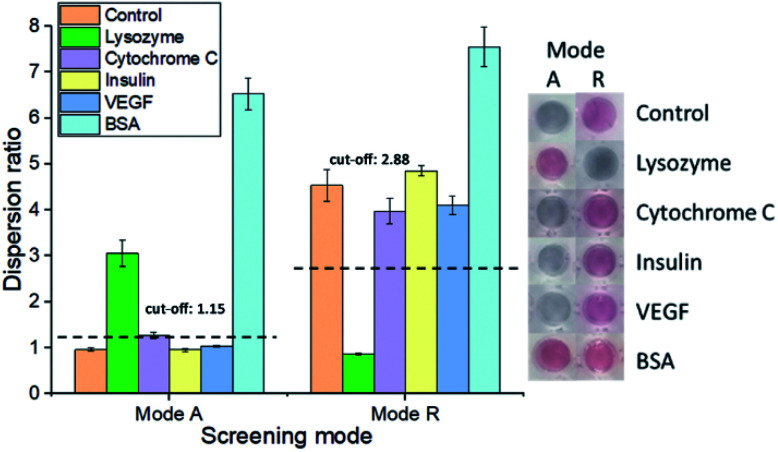
Selectivity performance of the proposed aptasensor towards lysozyme. The final concentrations of lysozyme and control proteins were tested at 180 and 375 nM for modes A and R, respectively. Inset: photos correspond to the samples.

To evaluate if our aptasensor is selective towards lysozyme (*M*_r_: 14.6 kDa; pI: 11), we have tested it against several control proteins with different sizes and isoelectric points, including cytochrome C (*M*_r_: 11.7 kDa; pI: 9.6), insulin (*M*_r_: 5.73 kDa; pI: 5.3), VEGF (*M*_r_: 19.3 kDa; pI: 6.6) and BSA (*M*_r_: 66.43 kDa; pI: 4.7). Samples containing lysozyme are dispersed in mode A and aggregated in mode R; whereas the others show the opposite results (Fig. 4 and S5†). An exception was observed for BSA samples, where the nanoparticles were dispersed in both modes, indicating that these bulky proteins may provide electro-steric stabilisation to the cysAuNPs. Notably, BSA has been widely used in passivating gold surfaces, particularly in positively charged nanomaterials such as CTAB-AuNRs.^38,39^ Avila *et al.* have also reported the preparation of AuNPs co-coated with both cysteamine and BSA, by mixing cysAuNPs with BSA.^40^ The high binding affinity of BSA towards metal surfaces could be attributed to its high-molecular-weight charges, as well as the prevalence of carboxylate, thiol, amine, and imidazole groups within the protein structure.^39,41–43^ Despite that, the positive error of BSA could be easily identified and eliminated based on its outlier dispersion ratios in both modes.

By calculating the averaged dispersion ratios of all the other control proteins ±3 S.D. for modes A and R, respectively, cut-off values could be set to distinguish lysozyme from the rest. Additionally, we have also tested the selectivity of the aptasensor against lysozyme in mixed protein sample. The selectivity of our aptasensor is attained since only lysozyme samples produced distinguishable results from other controls. The difference could even be observable by naked eyes under optimised condition (Fig. S6†).

### Comparison between modes A and R

As compared to mode R, mode A enables a more straightforward detection approach since the former requires careful screening to identify the CRC regime of DNA aptamer. However, the detection range of mode A is generally narrower, in accordance with other studies using cysAuNPs as aptasensor to detect melamine (1–24 nM)^15^ and 17β-estradiol (3.67–312.06 nM).^16^ This is attributed to the amount of aptamers available in mode A which is relatively lesser than that in mode R. Furthermore, as demonstrated in the selectivity test, mode A is also more susceptible to false positive result when bulky protein is assayed.

Having bimodal screening modes in a single assay has undoubtedly provided a complementary approach with greater versatility. On top of having the merit to choose the suitable screening mode, one can further validate the sensing results using the same batch of detection probes (*i.e.* cysAuNPs in this case). As demonstrated on our selectivity study, erroneous result can be eliminated with high accuracy. Furthermore, since modes A and R can be used for different concentration ranges of lysozyme, it provides a mean of tuneable dynamic range of detection.

## Conclusion

By manipulating the electrostatic interaction among DNA aptamer, target and cysAuNPs, we have rationally designed an aptasensor for lysozyme detection. While the concept of bimodal detection behaviour is relatively new, this study also shows the first demonstration of inverse sensitivity on spherical AuNPs. We envisage this work would encourage more studies to further explore and exploit the potential of cysAuNPs for different applications.

## Conflicts of interest

There are no conflicts to declare.

## Supplementary Material

RA-010-C9RA07930K-s001
