# Supplementary material for: Cysteamine-coated gold nanoparticles for bimodal colorimetric detection with inverse sensitivity: a proof-of-concept with lysozyme
Source: RSC Adv. 2020 Jan 6;10(2):1088–94. doi: 10.1039/c9ra07930k (PMC9047514; doi:10.1039/c9ra07930k)
Supplement: RA-010-C9RA07930K-s001 [file RA-010-C9RA07930K-s001.pdf]

Electronic Supplementary Material (ESI) for RSC Advances.
This journal is © The Royal Society of Chemistry 2019

Electronic Supplementary Information For

Cysteamine-Coated Gold Nanoparticles for Bimodal Colorimetric Detection with Inverse Sensitivity: A Proof-of-Concept with Lysozyme

Jing Luen Wai and Siu Yee New*

School of Pharmacy, University of Nottingham Malaysia, Jalan Broga, 43500, Semenyih, Selangor, Malaysia.

*Corresponding author:
E-mail: SiuYee.New@nottingham.edu.my

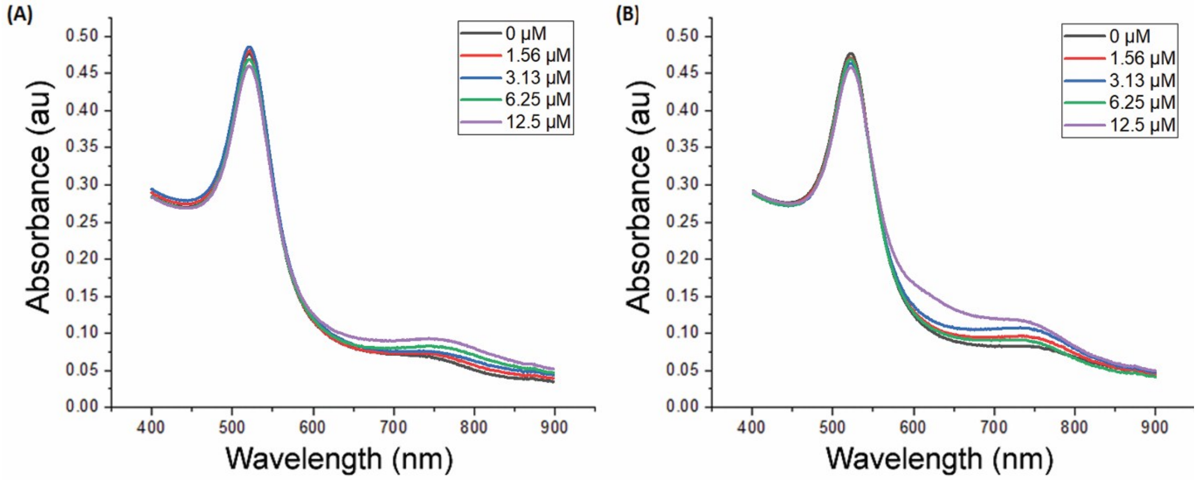

Figure S1: UV-Vis absorbance spectra of cysAuNPs in the presence of **(A)** mercaptoacetic acid and **(B)** acetylcysteine.

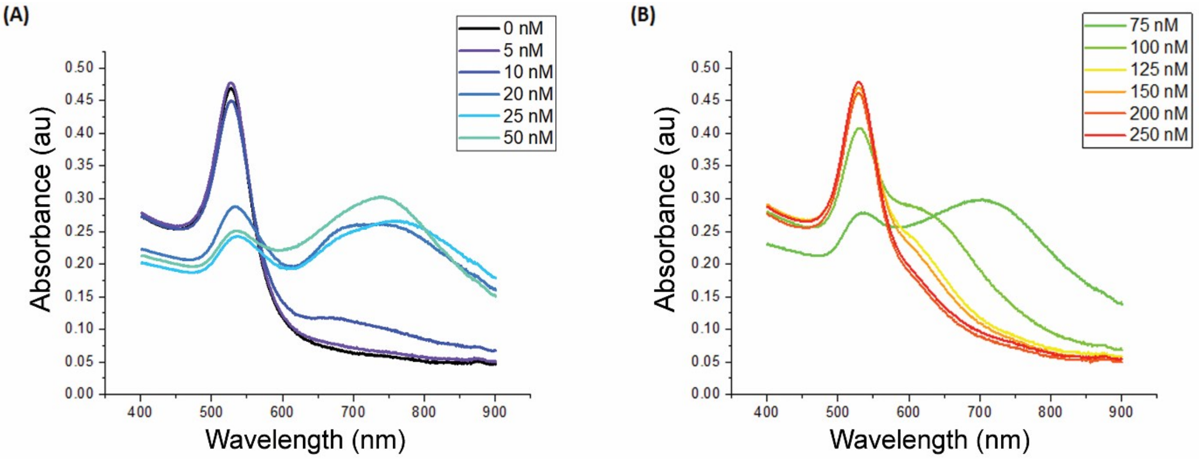

Figure S2: UV-Vis absorbance spectra of cysAuNPs incubated with different concentrations of LBA. **(A)** CysAuNPs aggregate as concentration of LBA increases from 0 to 50 nM. **(B)** CysAuNPs disperse as concentration of LBA increases from 75 to 250 nM.

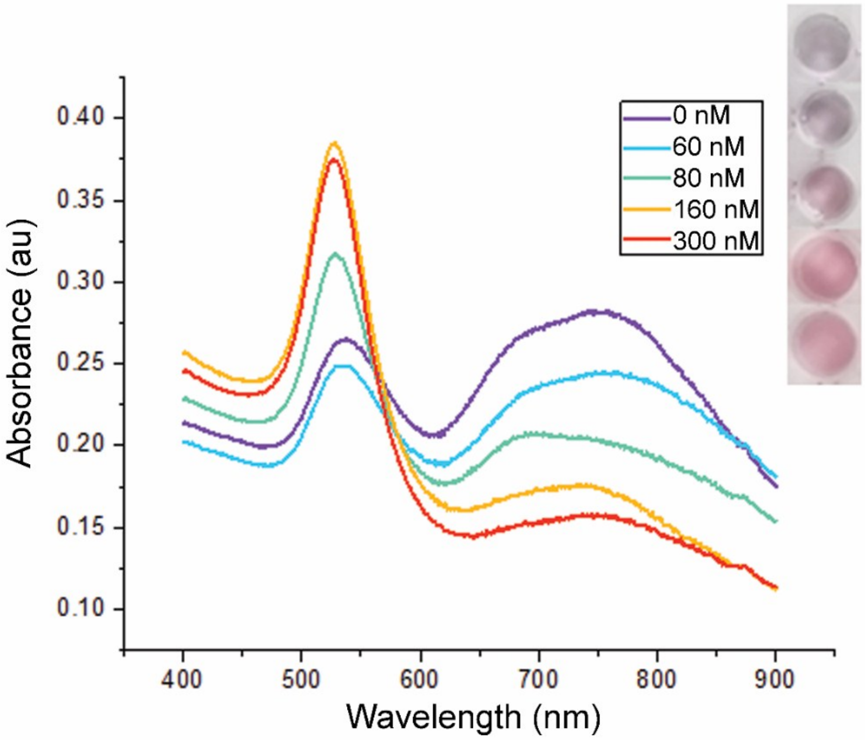

Figure S3: UV-Vis absorbance spectra of the aptasensor (mode A; consists of cysAuNPs and 25 nM of LBA) in detecting various concentrations of lysozyme. The spectra were selected to highlight the blue shift and re-establishment of LSPR band at 526 nm. Inset shows the progressive colour changes of cysAuNPs in the corresponding samples (top to bottom: 0 to 300 nM of lysozyme).

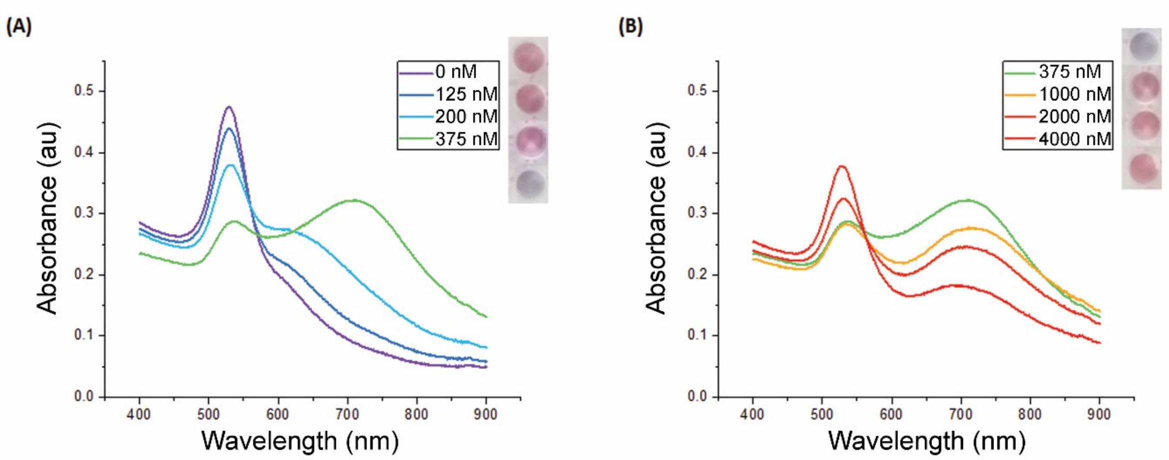

Figure S4: UV-Vis absorbance spectra of the aptasensor (mode R; consists of cysAuNPs and 150 nM of LBA) in detecting various concentrations of lysozyme. The spectra were selected to highlight **(A)** the increased aggregation and **(B)** redispersion of cysAuNPs. Inset shows the progressive colour change of cysAuNPs in the corresponding samples (top to bottom: **(A)** 0 to 375 nM of lysozyme; **(B)** 375 to 4000 nM of lysozyme).

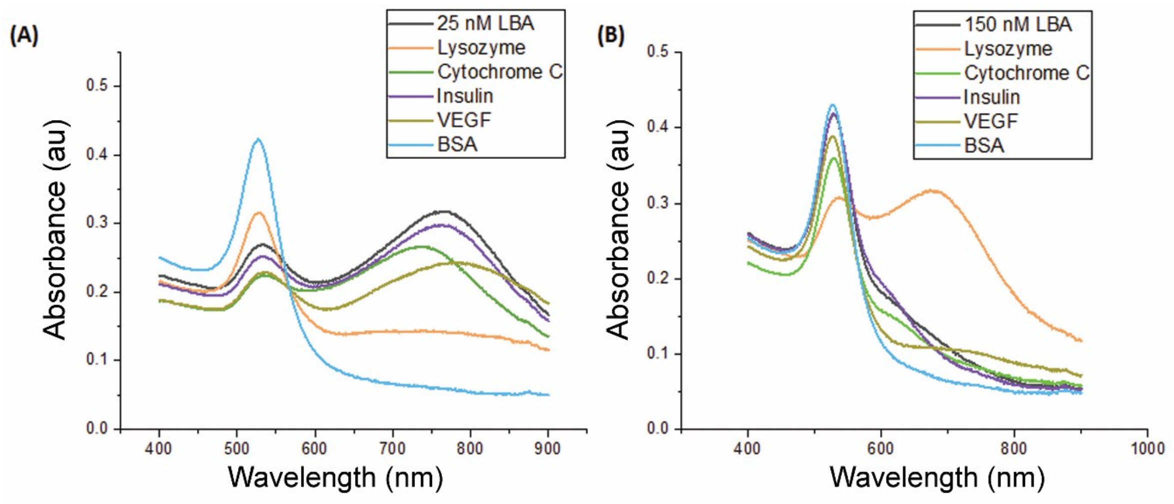

Figure S5: UV-Vis absorbance spectra of the aptasensor in **(A)** mode A and **(B)** mode R in the presence of lysozyme and control proteins.

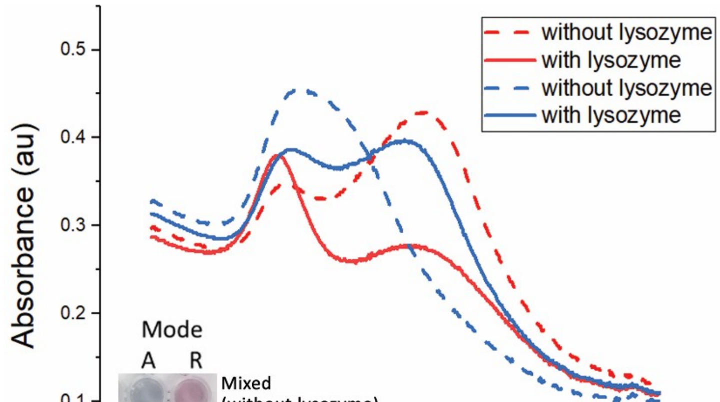

Figure S6. UV-Vis absorbance spectra of the aptasensor for lysozyme detection in mixed samples containing the control proteins (cytochrome C, insulin and VEGF) (red lines: mode A; blue lines: mode R). Mode A contains 540 nM of control proteins and 180 nM of lysozyme; whereas mode R contains 1125 nM of control proteins and 375 nM of lysozyme.

Table S1. Comparison of the reported colorimetric aptasensors for lysozyme detection.

Materials	LOD (nM)	Linear range (nM)
Polydiallyldimethylammonium chloride (PDDA), citAuNPs[1]	4.4 nM	4.4 – 200
cysAuNPs[2]	35	35– 1050
Human serum albumin (HSA)-modified citAuNPs[3]	50	100 – 1000
Core-shell Cu@AuNPs, 3,3,5,5-tetramethylbenzidine - hydrogen peroxide[4]	60	100– 1
Polydimethylsiloxane (PDMS)-AuNPs, Ag[5]	0.0069	0.69 – 69
cysAuNPs (this work)	2.29 (mode A)	37.5 – 180 (mode A)
	375 (mode R)	500 – 4000 (mode R)

References

1 X. Yao, X. Ma, C. Ding and L. Jia, *Microchim. Acta*, 2016, **183**, 2353–2359.

2 J. Su, W. Zhou, Y. Xiang, R. Yuan and Y. Chai, *Chem. Commun.*, 2013, **49**, 7659–7661.

3 Y.-M. Chen, C.-J. Yu, T.-L. Cheng and W.-L. Tseng, *Langmuir*, 2008, **24**, 3654–3660.

4 T. Lou, H. Qiang and Z. Chen, *Talanta*, 2017, **163**, 132–139.

5 W. Wang, W. Y. Wu, X. Zhong, W. Wang, Q. Miao and J. J. Zhu, *Biosens. Bioelectron.*, 2011, **26**, 3110–3114.
